# The Diagnostic Accuracy of Reduced-Dose CT of the Kidneys, Ureters, and Bladder in the Evaluation of Renal Colic

**DOI:** 10.7759/cureus.96882

**Published:** 2025-11-15

**Authors:** Osman Haji, Ling Paulina Gronczewska, Mohamed Nunow, Zehra Ali, Sarah A Davidson, Shyamala Manibalan

**Affiliations:** 1 General Medicine, NHS England, London, GBR; 2 General Medicine, West Suffolk Hospital, Bury St Edmunds, GBR; 3 General Surgery, Croydon Health Services NHS Trust, London, GBR; 4 Emergency, Croydon Health Services NHS Trust, London, GBR; 5 Geriatric Medicine, King's College Hospital NHS Foundation Trust, London, GBR

**Keywords:** acute renal colic, ctkub, ct scan sensitivity, low-dose computed tomography, renal calculi

## Abstract

Non-contrast CT of the kidneys, ureters, and bladder (CT KUB) is the reference standard for suspected renal colic, but radiation exposure is a concern. Reduced-dose CT KUB protocols have been developed to minimise risk, and multiple systematic reviews have assessed their diagnostic accuracy. Our objective was to evaluate the diagnostic performance of low-dose (LD) and ultra-LD (ULD) CT KUB for suspected urolithiasis and to evaluate the methodological quality of systematic reviews written on this topic. A systematic search in June 2025 was conducted on MEDLINE, Embase, PubMed, and the Cochrane Library from inception until the time of search, yielding 1,357 results, with 1,318 remaining after de-duplication. Three systematic reviews were eligible. Data were extracted on study scope, populations, definitions of LD/ULD dose, accuracy outcomes, and review quality. Risk of bias was appraised using risk of bias in systematic reviews (ROBIS). The three included reviews synthesised between six and 12 primary diagnostic accuracy studies (sample sizes: 903-1,529 participants) and were published in the mid-to-late 2010s. Pooled estimates from one review demonstrated high diagnostic performance of LD and ULD CT KUB (ULD: sensitivity 95%, specificity 97%; LD: sensitivity 92%, specificity 97%). The remaining reviews provided narrative syntheses, consistently reporting sensitivities and specificities above 85%, though diagnostic performance was reduced in obese patients and for stones <3 mm. All reviews highlighted the potential to detect alternative diagnoses. ROBIS identified one review at low risk of bias, one at moderate risk (due to heterogeneous reference standards and language restrictions), and one at high risk (no primary study quality appraisal). Existing systematic reviews consistently support reduced-dose CT KUB as highly accurate for diagnosing urolithiasis, with performance approaching standard-dose CT, though limitations exist in obese patients and for very small stones. Methodological quality of the reviews was variable. Further high-quality reviews using standardised methods are warranted to refine estimates and assess generalisability.

## Introduction and background

Renal colic is a common presentation to emergency departments worldwide [1] and is most frequently caused by urolithiasis [2]. The lifetime prevalence of kidney stones is increasing and is typically higher in developed countries, with incidence rising due to dietary, metabolic, and environmental risk factors [3]. Acute episodes are characterised by sudden, severe flank pain, often associated with haematuria, nausea, and urinary tract symptoms [4]. Prompt and accurate diagnosis is essential [5], not only to confirm the presence of calculi but also to exclude important alternative pathologies, such as appendicitis, diverticulitis, or other causes of an acute abdomen, which may mimic renal colic clinically [6]. Imaging is therefore central to the diagnostic work-up of suspected stone disease [7].

Non-contrast computed tomography of the kidneys, ureters, and bladder (CT KUB) has become the reference standard imaging modality for suspected renal colic [8]. Compared with traditional modalities, such as intravenous urography (IVU), plain abdominal radiography, or ultrasound, CT KUB offers superior sensitivity and specificity, rapid acquisition, and the ability to detect both stones and alternative diagnoses [9]. Reported sensitivities and specificities for standard-dose CT in the detection of urinary tract stones approach 95-100%, with high reproducibility across different practice settings. For these reasons, guidelines from the American Urological Association (AUA) and the European Association of Urology (EAU), endorse CT KUB as first-line imaging in adults with suspected urolithiasis [10].

Despite its diagnostic advantages, CT KUB is associated with a non-trivial radiation burden. Standard-dose CT protocols for renal colic typically deliver effective doses around 10 mSv [7], though doses may vary depending on scanner technology, acquisition parameters, and patient body habitus [11]. While this level of exposure is modest compared with some oncological CT protocols, it is of concern in the context of renal colic because the condition frequently affects younger adults, who have longer lifespans during which radiation-induced malignancies could manifest [12]. Furthermore, stone disease is recurrent in up to 50% of patients within 10 years [13], raising the prospect of repeated imaging and cumulative exposure. In addition to this, it is not uncommon that CT KUBs often "over-scan" patients, with imaging commonly going beyond recommended limits (the upper pole of the kidney on one end and the pubic symphysis on the other end) [14]. The risk-benefit balance of CT KUB is therefore less straightforward in this group [15], particularly when alternative modalities with lower or no radiation are available, even if they sacrifice some diagnostic accuracy.

Efforts to mitigate radiation exposure have led to the development and increasing adoption of reduced-dose CT protocols [16]. Advances in scanners now allow substantial dose reductions while maintaining acceptable diagnostic performance [17]. Reduced-dose CT is typically categorised into low-dose (LD) and ultra-LD (ULD) protocols [18], though definitions vary across studies. LD CT KUB is often considered to deliver effective doses in the 2-4 mSv range, whereas ULD protocols may achieve sub-2 mSv exposures, approaching the level of a plain abdominal radiograph [19]. The challenge lies in ensuring that these dose reductions do not compromise sensitivity for clinically significant stones or the ability to detect alternative diagnoses.

A growing number of diagnostic accuracy studies over the past two decades have evaluated LD and ULD CT KUB against standard-dose reference protocols. While many demonstrate high sensitivity and specificity, variability exists in certain cases, particularly with obese patients [20]. The clinical significance of missed small stones remains debated, as they often pass spontaneously [21], but under-detection in obese individuals is more concerning, given the increasing prevalence of obesity and its association with urolithiasis [22]. In addition, the ability of reduced-dose protocols to identify incidental but potentially life-threatening alternative diagnoses remains uncertain.

To synthesise this growing evidence, a few systematic reviews have been conducted in the 2010s, each addressing different aspects of reduced-dose CT. Approaches taken include quantitative meta-analyses, pooling sensitivity and specificity estimates across dose ranges, as well as narrative syntheses of diagnostic performance and technical considerations. However, methodological limitations are evident. Reviews vary in their eligibility criteria, search comprehensiveness, handling of heterogeneity, and application of risk-of-bias assessment tools. Other differences include whether they restricted inclusion to English-language studies, whether they assessed publication bias, and how they conducted quality appraisal of primary studies.

In this context, clinicians, radiologists, and policymakers face a complex evidence landscape. While the general message is consistent - that reduced-dose CT KUB is highly accurate and clinically viable - uncertainty remains regarding the strength and reliability of the evidence base. For example, should reduced-dose CT be recommended universally as first-line imaging in suspected renal colic, or only in selected populations such as non-obese adults? How robust are the pooled sensitivity and specificity estimates, given the methodological heterogeneity among reviews? And to what extent might publication bias or selective reporting inflate diagnostic accuracy estimates?

Systematic reviews of systematic reviews, sometimes termed “overviews” or “umbrella reviews,” have emerged as a methodological approach to address such questions. By collating and appraising evidence from multiple existing reviews, overviews can provide a higher-level synthesis, highlight areas of consensus or discrepancy, and evaluate the methodological quality of the reviews themselves. For topics such as reduced-dose CT, where multiple reviews already exist but vary in scope and quality, an overview can clarify the current state of evidence and provide practical recommendations for clinicians and guideline developers.

A key component of any overview is the critical appraisal of included systematic reviews. Several appraisal tools exist, most notably AMSTAR-2 (A MeaSurement Tool to Assess Systematic Reviews, version 2) and ROBIS (risk of bias in systematic reviews). AMSTAR-2 is widely recognised and assesses methodological quality across 16 domains but was primarily developed for intervention reviews, and questions remain about its applicability to diagnostic accuracy reviews [23]. ROBIS, in contrast, was specifically designed to evaluate risk of bias in systematic reviews across different domains, including diagnostics [24], and may therefore offer a more appropriate lens for evaluating the current evidence on reduced-dose CT.

Against this background, the present study aimed to conduct a systematic review of systematic reviews evaluating the diagnostic accuracy of LD and ULD CT KUB for suspected renal colic. Our objectives were threefold: first, to summarise the findings of existing reviews regarding sensitivity, specificity, and clinical utility of reduced-dose CT KUB; second, to appraise the methodological quality and risk of bias of these reviews using validated tools; and third, to identify gaps and limitations in the current evidence base that warrant further research. By addressing these objectives, we seek to provide clinicians, radiologists, and policymakers with a clear and balanced synthesis of the evidence, supporting informed decision-making in the imaging of suspected renal colic.

## Review

Methods

This systematic review of systematic reviews was conducted in accordance with the Preferred Reporting Items for Systematic Reviews and Meta-Analyses (PRISMA) statement and methodological guidance for overviews of reviews. A formal protocol was developed a priori, specifying eligibility criteria, search strategy, and planned approaches for data extraction and quality appraisal.

Eligibility Criteria

We included systematic reviews that evaluated the diagnostic accuracy of LD or ULD non-contrast CT KUB for the diagnosis of urolithiasis in adults with suspected renal colic. Reviews were eligible if they reported a systematic search strategy, synthesised primary diagnostic accuracy studies, and presented sensitivity, specificity, or other diagnostic performance outcomes. Both meta-analytical and narrative systematic reviews were eligible. We excluded non-systematic narrative reviews, conference abstracts, editorials, and reviews not reporting original synthesis of primary studies. There were no restrictions based on publication date, but only reviews published in English were included.

The index test of interest was LD or ULD CT KUB, with dose thresholds defined according to each review’s criteria (commonly <3-4 mSv for LD, and <2 mSv for ULD). The reference standard was standard-dose CT or, where applicable, composite standards including endoscopic stone confirmation, follow-up imaging, or clinical outcome. The primary outcomes were diagnostic sensitivity and specificity for the detection of urinary tract calculi. Secondary outcomes included diagnostic accuracy in subgroups (e.g. obese patients, small stones) and the ability to identify alternative diagnoses.

Information Sources and Search Strategy

We systematically searched four electronic databases: MEDLINE (via Ovid), Embase (via Ovid), the Cochrane Library, and PubMed. The search covered all records from database inception to June 2025. Search terms combined synonyms and controlled vocabulary for “computed tomography”, “CT KUB”, “low-dose”, “ultra-low-dose”, “renal colic”, “urolithiasis”, and “systematic review” (Appendix, Tables 3-6). No date restrictions were applied, but the search was limited to English-language publications. Reference lists of included reviews were screened to identify additional eligible studies.

Study Selection

All retrieved citations were imported into a reference management database (Zotero), and duplicates were removed. A total of 1,357 records were identified, of which 39 were duplicates, leaving 1,318 unique records. Titles and abstracts were independently reviewed against the eligibility criteria by two reviewers, with disagreements to be resolved by discussion and then seeking the opinion of a senior reviewer. Full texts were then retrieved for potentially relevant records and assessed.

Data Extraction

Data were extracted independently by two reviewers using a piloted extraction form. Extracted variables included review characteristics (authors, year, journal, country), search strategy details (databases searched, date ranges, language restrictions), eligibility criteria, definitions of LD/ULD CT, number and characteristics of included primary studies, sample sizes, reference standards, and diagnostic accuracy outcomes (sensitivity, specificity, pooled estimates if available). We also extracted whether reviews assessed alternative diagnoses, patient subgroups, or radiation dose estimates.

Risk of Bias and Methodological Quality Assessment

The methodological quality of included systematic reviews was appraised using the ROBIS tool, which is specifically designed to assess risk of bias in systematic reviews across four domains: study eligibility criteria; identification and selection of studies; data collection and study appraisal; and synthesis and findings. Each domain was rated as low, high, or unclear risk of bias, with an overall judgement assigned for each review. Quality assessments were undertaken independently, with discrepancies resolved through discussion and consensus.

Data Synthesis

Given the small number of eligible systematic reviews and variability in their methods and outcomes, we performed a descriptive synthesis. Results were summarised narratively, with emphasis on reported diagnostic performance of LD and ULD CT KUB, methodological quality, and risk of bias of each review. Where available, pooled sensitivity and specificity estimates from meta-analyses were reported alongside ranges from narrative reviews. Particular attention was paid to differences in review scope, definitions of dose categories, reference standards, and quality appraisal methods, as these factors contribute to heterogeneity.

Results

Search Results

**Figure 1:**
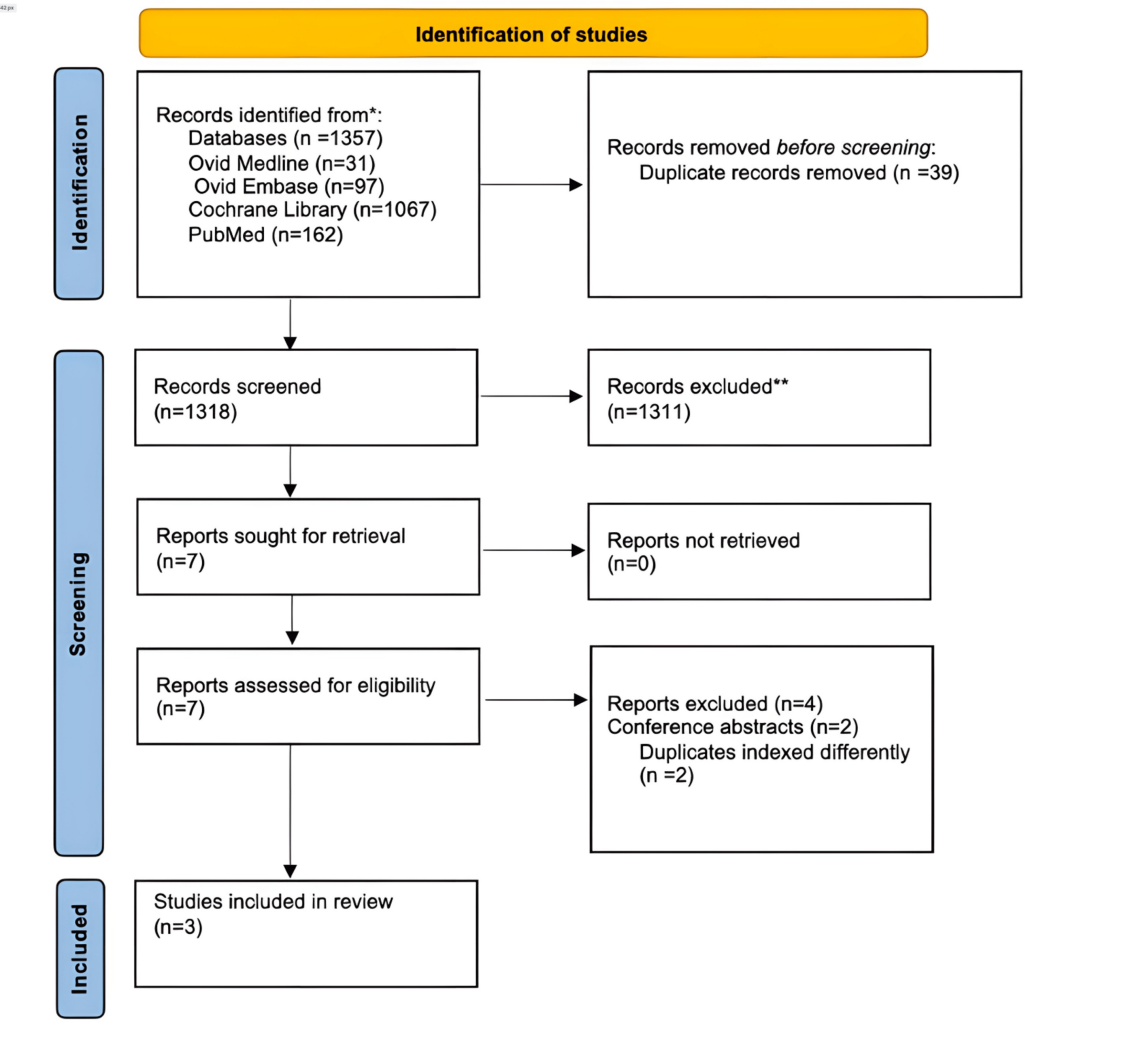
PRISMA flow diagram PRISMA: Preferred Reporting Items for Systematic Reviews and Meta-Analyses

The database search identified 1,357 records. After removal of 39 duplicates, 1,318 unique titles and abstracts were screened. Following full-text review, three systematic reviews met the eligibility criteria and were included in this overview. A PRISMA flow diagram of study selection is provided in Figure 1.

Characteristics of the Included Reviews

The three included reviews were published between 2014 and 2018 and collectively synthesised between six and 12 primary diagnostic accuracy studies, with sample sizes ranging from 903 to 1,529 participants. All reviews focused on adults presenting with suspected renal colic, evaluating LD or ULD non-contrast CT KUB against standard-dose CT or composite reference standards. Definitions of LD and ULD protocols varied, most commonly <3-4 mSv for LD and <2 mSv for ULD. All reviews reported sensitivity and specificity as primary outcomes and discussed diagnostic challenges in subgroups such as obese patients or those with very small stones.

The results and risk of bias assessments are documented in Tables 1-2.

**Table 1:** Study characteristics and key findings

Paper	Databases searched	Inclusion criteria	Number of studies included	Participants	Population characteristics	Findings	Quality/risk of bias
Drake et al., 2014 (Indian J Urol) [25]	Cochrane Library, MEDLINE, EMBASE, DARE (1990–Oct 2012); English only	Prospective adult (non-pregnant) studies using low-dose non-contrast CT KUB (<3 mSv) with reference standard (standard-dose CT or composite). Excluded cadaveric/simulated/contrast/pregnancy/unclear standard	6	903	Adults aged 15–90; stone prevalence 36–88%; additional pathologies 5–16%; doses 0.5–2.8 mSv	Sensitivity 90–97%, specificity 86–100%. Misses <3 mm stones; accuracy lower in BMI>30. Grade A recommendation for non-obese first-line use	QUADAS used; 1 Level 1A, 2 Level 1B studies; modest quality, spectrum/recruitment bias; no cost analyses
Rob et al., 2017 (Clin Radiol) [26]	PubMed, MEDLINE, EMBASE, Ovid (Jan 1995–Sep 2015); English only; PRISMA/Cochrane compliant	Prospective adult studies with standard, low (<3.5 mSv), or ultra-low (≤1.9 mSv) non-contrast CT KUB; excluded retrospective/pregnancy/children/cadaveric	7	1104	Countries: South Korea, Switzerland, Belgium, Germany, USA; ULD 0.5–1.9 mSv; stone prevalence 36–73%; alternative diagnoses 12–15%	Sensitivity 90–100%, specificity 86–100%. May underdetect stones <3 mm or in BMI>30	Two reviewers; LD and ULD defined consistently; emphasis on dose–noise trade-offs; limited heterogeneity analysis
Rodger et al., 2018 (Urol Int) [27]	MEDLINE, EMBASE, Cochrane CENTRAL, Google Scholar, PubMed, individual urology journals (to Aug 2017); no language limits	Studies comparing LD (<3.5 mSv) or ULD (<1.9 mSv) CT KUB with reference standard (standard-dose CT or physical stone proof). Excluded studies without reference standard	12	1,439–1,529 (discrepancy between abstract and text)	Publications 2001–2015; countries: Germany, South Korea, Belgium, Switzerland, France, China, UK, USA, Kenya; LD 2.1–4.5 mSv; ULD 0.48–1.9 mSv.	LD: sens 90–98%, spec 88–100%; pooled sens 91.9%, spec 97.2%, accuracy 94.3%. ULD: sens 72–99%, spec 86–100%; pooled sens 95.2%, spec 96.9%, accuracy 95.5%.	High overall quality (QUADAS-2); consecutive recruitment; some heterogeneity in dose protocols; iterative reconstruction reduces noise; caution re limited alternative diagnosis detection

**Table 2:** Risk of bias assessments ROBIS: risk of bias in systematic reviews

Paper	Domain 1: Eligibility criteria	Domain 2: Identification/Selection	Domain 3: Data collection/Appraisal	Domain 4: Synthesis/Findings	Overall ROBIS risk	Key Rationale
Drake et al., 2014 (Indian J Urol) [25]	Low	High	Low	High	Moderate	Used QUADAS and Cochrane DTA; English-only restriction and limited search detail
Rob et al., 2017 (Clin Radiol) [26]	High	High	High	High	High	No formal bias tool, English-only search, no grey literature, and unclear screening totals
Rodger et al., 2018 (Urol Int) [27]	Low	Low	Low	Low	Low	Comprehensive multi-database search, QUADAS-2 used, dual extraction; minor missing publication bias analysis

Rodger et al. [27]: They undertook a systematic review and meta-analysis to determine the diagnostic accuracy of LD and ULD CT KUB in patients presenting with suspected renal colic. They included 12 primary diagnostic accuracy studies encompassing a broad range of scanners and patient populations. Dose thresholds were defined pragmatically, with LD protocols generally delivering 2.1-4.5 mSv and ULD protocols 0.48-1.9 mSv. Standard-dose CT served as the reference.

The central results of the review are the pooled estimates of sensitivity and specificity. For ULD CT KUB, the meta-analysis demonstrated a pooled sensitivity of 95.2% (95% CI: 93.7-96.4%) and a pooled specificity of 96.9% (95% CI: 95-98%). For LD CT KUB, pooled sensitivity was slightly lower at 91.9% (95% CI: 87.8-95.0%), while pooled specificity remained very high at 97.2% (95% CI: 94-99%). These findings indicate that both LD and ULD CT protocols retain excellent diagnostic accuracy, approaching the performance of standard-dose CT, which is typically quoted at 95-100% for both sensitivity and specificity.

The review also presented summary receiver-operating characteristic (SROC) curves, which demonstrated clustering of individual studies in the upper-left quadrant, reinforcing high overall accuracy. The area under the SROC curves showed clustering of studies near the upper-left corner, indicating high overall accuracy.

Despite these strong pooled results, the authors identified notable heterogeneity, particularly for specificity in the ULD group (I² ≈ 61.5%). They attributed this to differences in scanner technology, patient selection, and the choice of reference standards across primary studies. Some studies employed surgical confirmation or spontaneous stone passage as proof of disease, while others relied solely on comparison with standard-dose CT. Such variability complicates direct comparisons and likely inflates heterogeneity statistics.

No formal subgroup analyses or meta-regressions were undertaken to explore sources of heterogeneity further, but the authors acknowledged that patient characteristics, protocol optimisation, and definitions of “low” and “ultra-low” dose could all contribute.

Several clinically important subgroup issues emerged from the included studies and were synthesised narratively in the review. Across multiple studies, diagnostic performance was attenuated in obese patients. Increased body mass index (BMI >30) was associated with higher image noise and reduced sensitivity, particularly for small stones, and in some cohorts, this effect was sufficient to reduce confidence in ULD scans.

A second consistent finding was the reduced detection rate for very small stones (<3 mm). While pooled estimates still showed sensitivities above 90%, some individual studies reported missed calculi at these sizes. The clinical importance of such stones is debated, as they are more likely to pass spontaneously, but their omission may influence acute management decisions.

An important advantage of CT imaging for renal colic, beyond stone detection, is the ability to identify alternative or incidental pathologies. Although not the primary focus of the meta-analysis, Rodger et al. [27] reported that several included studies demonstrated that LD and ULD protocols could still detect significant alternative diagnoses, such as appendicitis, diverticulitis, and abdominal aortic aneurysm. The review concluded that, while the ability to detect alternative diagnoses may be somewhat reduced at the lowest dose settings, diagnostic confidence remained clinically useful in most settings.

By aggregating 12 studies, the review provided the most comprehensive quantitative synthesis of reduced-dose CT KUB available at the time. The pooled sensitivity and specificity estimates were both comfortably above 90%, with narrow confidence intervals in the ULD group reflecting consistency of results. These data strongly support the proposition that radiation dose can be markedly reduced without significant compromise in diagnostic accuracy, at least in average-sized adult patients.

Nevertheless, the authors were clear about limitations. Heterogeneity in protocol definitions and imaging parameters means that “ULD” in one study may not be equivalent to another. Reference standards varied, raising potential for incorporation bias in studies that used standard-dose CT alone. Few studies reported detailed subgroup analyses, making it difficult to quantify exactly how diagnostic performance changes with BMI, stone size, or scanner type. Furthermore, relatively few of the included studies had very large sample sizes, which limits the precision of subgroup findings.

The review team applied QUADAS-2 to assess methodological quality of the 12 included primary studies. The risk-of-bias graphs highlighted common issues in patient selection (unclear or non-consecutive enrolment), blinding of index test interpretation, and heterogeneity of reference standards. Flow and timing were also inconsistently reported, raising the possibility that disease progression or treatment could have influenced results between CT examinations and confirmation of diagnosis.

At the level of the review itself, strengths included duplicate data extraction, formal quality appraisal, and meta-analysis with SROC presentation. Weaknesses included limited reporting of the literature search strategy, absence of a list of excluded studies, and no formal assessment of publication bias. The authors did not explore the impact of study quality on pooled estimates, which limits confidence in the robustness of the meta-analysis.

Rodger et al. [27] concluded that both LD and ULD CT KUB are highly accurate for the diagnosis of urinary tract stones and can be considered viable alternatives to standard-dose CT in most clinical settings. They emphasised that diagnostic performance is slightly reduced in obese patients and for very small calculi, but overall sensitivity and specificity remained well above 90%. Importantly, reduced-dose protocols also retained the capacity to detect clinically significant alternative diagnoses.

From a methodological standpoint, this review provided the strongest quantitative evidence base among available systematic reviews, with robust pooled estimates and a clear conclusion favouring reduced-dose CT. However, limitations in search reporting, heterogeneity, and incomplete integration of risk-of-bias assessments temper the certainty of the findings. For clinicians and guideline developers, the results strongly suggest that substantial dose reduction is feasible without materially compromising diagnostic utility, though caution is warranted in high-BMI patients and when exclusion of very small stones is clinically critical.

Rob et al. [26]: They published a systematic review in 2017 entitled “Ultra-Low-Dose, Low-Dose, and Standard-Dose CT KUB: Is There a Difference?”. Their aim was to determine whether reducing radiation exposure in CT KUB compromises diagnostic accuracy in patients presenting with suspected renal colic.

The review synthesised seven prospective diagnostic accuracy studies, involving a total of 1,104 participants recruited between 2002 and 2015. All studies evaluated adult patients with acute flank pain and clinical suspicion of urolithiasis. Reduced-dose categories were defined as ULD ≤1.9 mSv and LD <3.5 mSv. Standard-dose CT served as the reference in most studies, with some also incorporating clinical confirmation or stone passage. The mean male-to-female ratio across studies was approximately 3:2, reflecting the recognised male predominance of stone disease.

Across the included studies, diagnostic performance remained high at both LD and ULD levels. Sensitivities ranged from 90% to 100%, and specificities from 86% to 100%. Importantly, no included study reported sensitivity below 90%, suggesting that reduced-dose CT protocols consistently deliver clinically acceptable accuracy.

The review did not undertake quantitative pooling due to methodological heterogeneity, but the narrative synthesis emphasised that the majority of ULD protocols (<2 mSv) achieved performance metrics similar to LD and standard-dose imaging. For example, in studies employing ULD CT with doses as low as 0.5-0.7 mSv, diagnostic accuracy for urolithiasis remained within 2-3 percentage points of standard-dose comparators. This reinforces the feasibility of significant dose reductions without major compromise in sensitivity or specificity.

A consistent theme across studies was reduced diagnostic performance in specific patient groups. In obese patients (BMI >30), image noise was increased, and sensitivity declined modestly. While stone detection rates remained above 85%, the relative decrement compared with non-obese patients was clinically relevant, particularly in ULD protocols. This finding aligns with the known technical challenge of dose reduction in larger body habitus, where greater photon penetration is required to achieve diagnostic image quality.

A second limitation involved very small stones (<3 mm). Several studies reported reduced detection rates at these sizes, with sensitivity occasionally dropping into the mid-80% range. Although the clinical significance of such small stones is debatable - many pass spontaneously - failure to identify them can complicate clinical decision-making, especially in symptomatic patients. Rob et al. [26] emphasised this limitation and recommended caution in interpreting ULD CT scans in patients where exclusion of small stones is clinically critical.

One of the most clinically relevant contributions of the review was its synthesis of data on alternative diagnoses. Across the included studies, alternative or incidental pathologies were detected in approximately 12-15% of patients undergoing ULD CT. These included diverticulitis, appendicitis, ovarian pathology, and vascular abnormalities. The ability to identify such conditions is a key advantage of CT KUB compared with ultrasound or radiography, and the review concluded that ULD CT retains this important feature. Although diagnostic confidence may be slightly reduced at the very lowest dose protocols, clinically significant alternative diagnoses were not systematically missed.

The review highlighted that ULD protocols can reduce radiation dose to levels approaching those of a plain abdominal radiograph (0.5-1.9 mSv) while maintaining high diagnostic performance. This represents a significant reduction compared with standard-dose CT (8-10 mSv). The clinical importance of this lies in the demographics of stone disease: patients are often young and recurrent stone formers, for whom cumulative radiation exposure is a significant long-term concern.

Unlike some other reviews in this area, Rob et al. [26] did not report a formal structured risk-of-bias assessment of the included primary studies (e.g. using QUADAS-2). While they performed duplicate data extraction and adhered to PRISMA guidance, the absence of quality appraisal is a notable limitation, as it prevents readers from judging how methodological flaws in primary studies may have influenced the findings. In addition, the review limited inclusion to English-language publications, introducing a risk of language bias. On the positive side, the review focused exclusively on prospective studies, which strengthens internal validity relative to retrospective designs.

Rob et al. [26] concluded that both LD and ULD CT KUB are highly sensitive and specific for the detection of urolithiasis and that reducing radiation exposure to below 2 mSv is feasible without substantial diagnostic compromise. They highlighted the consistent ability of ULD CT to detect clinically important alternative diagnoses, an essential consideration in acute flank pain. The authors cautioned, however, that diagnostic performance may be reduced in obese patients and for very small stones and that clinicians should interpret negative ULD CT findings with caution in these contexts.

Overall, this review reinforced the clinical viability of reduced-dose CT, particularly ULD protocols, and provided compelling evidence that diagnostic accuracy remains above 90% in most populations. The absence of formal risk-of-bias assessment of primary studies tempers confidence in the findings, but the consistency of results across seven prospective studies provides reassurance that the conclusions are broadly reliable.

Drake et al. [25]: This was a systematic review published in 2014 entitled “Should Low-Dose CT KUB Be the New Investigation of Choice for Suspected Renal Colic? A Systematic Review.” The review synthesised evidence on the diagnostic accuracy of LDCT for patients presenting with suspected renal colic.

The review included six prospective diagnostic accuracy studies, with a combined total of 903 participants. All were adult patients presenting with acute flank pain. LDCT was defined as delivering an effective radiation dose of <3 mSv, with comparisons made against standard-dose CT or, in some cases, composite reference standards including stone passage, surgical confirmation, or follow-up imaging. Stone prevalence in the included studies ranged from 36% to 87.9%, reflecting typical real-world emergency department populations.

Across the six included studies, the diagnostic performance of LDCT remained consistently high, with both sensitivity and specificity generally reported above 90%. Although the review did not provide pooled estimates due to heterogeneity, the consistency of results across studies provided confidence in the reliability of LDCT as a diagnostic test.

One of the larger included studies reported a sensitivity of 96% and a specificity of 97%, almost indistinguishable from standard-dose CT. Other studies demonstrated slightly lower sensitivities, typically in the 92-94% range, but with specificities still approaching 95-100%. The range of results demonstrated that, even with significant reductions in radiation dose, diagnostic accuracy remained well within clinically acceptable thresholds.

The review highlighted that diagnostic performance was particularly challenged in the detection of very small calculi (<3 mm). Several studies noted that LDCT sometimes failed to identify stones of this size, largely due to increased image noise at reduced dose levels. However, Drake et al. [25] pointed out that the clinical relevance of these very small stones is uncertain, as most pass spontaneously without intervention. Thus, while LDCT may under-detect them, the impact on patient management may be limited in many cases.

A second limitation repeatedly observed in the included studies was reduced sensitivity in obese patients (BMI >30). In these individuals, LDCT images were noisier, and small calculi were more easily obscured. Sensitivity was reduced in obese patients (BMI>30), although the exact magnitude was not explicitly stated. Drake et al. [25] concluded that, while LDCT remains useful, clinicians should be aware of this limitation and may need to consider alternative imaging strategies in high-BMI populations.

An important clinical consideration addressed by the review was the ability of LDCT to detect alternative diagnoses. Although not consistently reported in all studies, several provided data showing that reduced-dose protocols retained diagnostic capability for non-stone pathology. Examples included appendicitis, diverticulitis, gynaecological conditions, and vascular abnormalities. The review emphasised that this is a critical advantage of CT imaging over ultrasound or plain radiography and that LDCT largely preserved this benefit despite dose reduction.

Radiation doses across the included LDCT protocols were consistently below 3 mSv, with some approaching 1.5-2 mSv. Compared to typical standard-dose CT exposures of 8-10 mSv, this represents a reduction of approximately two-thirds. Drake et al. [25] underscored the importance of this finding given the demographics of stone disease: patients are often in early adulthood and prone to recurrent episodes requiring repeated imaging. By reducing dose while maintaining accuracy, LDCT offers a safer long-term diagnostic strategy.

Drake et al. [25] concluded that LDCT is both safe and effective for the diagnosis of urolithiasis in suspected renal colic. With sensitivities and specificities consistently above 90%, they considered it a viable first-line imaging modality, particularly in non-obese patients. They cautioned that accuracy may be reduced in obese individuals and that very small stones may be missed, but argued that these limitations are outweighed by the radiation-sparing benefits. Importantly, they noted that LDCT also frequently identifies alternative pathologies, ensuring comprehensive diagnostic coverage in acute flank pain presentations.

The review applied the original QUADAS tool to appraise primary study quality. The appraisal identified common limitations, including variability in reference standards (some relying solely on CT comparison, others using composite outcomes), potential spectrum bias from high stone prevalence cohorts, and incomplete reporting of patient selection. Nevertheless, the decision to limit inclusion to prospective studies improved methodological consistency.

At the review level, strengths included a clearly defined search across multiple databases (MEDLINE, Embase, Cochrane, DARE) up to October 2012, dual-reviewer screening, and independent data extraction. Limitations included restriction to English-language publications, lack of meta-analysis due to heterogeneity, and absence of publication bias assessment. As a result, the review was judged at moderate risk of bias, with the main concerns stemming from language restriction and variable reference standards across studies.

Drake et al.’s [25] review was among the earliest systematic syntheses supporting LDCT KUB as a realistic alternative to standard-dose CT. By demonstrating consistently high diagnostic accuracy in six prospective cohorts, it provided compelling evidence that doses can be substantially reduced without sacrificing clinical utility. While methodological limitations temper the certainty of conclusions, the review’s findings remain influential and have been reinforced by subsequent meta-analyses. For clinicians, the key message is that LDCT KUB should be considered a first-line imaging test for suspected renal colic, with the caveat that diagnostic performance may be compromised in obese patients and for stones <3 mm.

Discussion

This overview of systematic reviews synthesised the best available secondary evidence on the diagnostic performance of reduced-dose CT KUB in patients with suspected renal colic. Across three included systematic reviews, drawing on between six and twelve primary diagnostic accuracy studies each, the results consistently demonstrated that both LD (typically <3-4 mSv) and ULD (<2 mSv) CT protocols achieve sensitivities and specificities exceeding 90% for the detection of urinary tract stones. Where meta-analysis was undertaken, pooled estimates for ULD CT approached 95% sensitivity and 97% specificity, values only marginally below those traditionally reported for standard-dose CT. Narrative syntheses echoed these findings, reporting similarly high diagnostic accuracy across diverse patient cohorts.

Importantly, all reviews noted that diagnostic performance is somewhat reduced in particular clinical contexts. Obese patients represent the most consistent challenge, with greater image noise at reduced doses contributing to decreased sensitivity. Similarly, very small calculi (<3 mm) were occasionally missed, reflecting the technical limitations of dose reduction. The clinical impact of these limitations is nuanced: while very small stones often pass spontaneously and may not alter management, failure to detect stones in obese patients can compromise diagnostic confidence. These findings suggest that reduced-dose CT KUB is highly effective for the majority of patients but should be interpreted with caution in specific subgroups.

Another consistent finding was the ability of LD and ULD CT to detect alternative or incidental diagnoses, reported in 10-15% of patients in some studies. This capacity is a major strength of CT compared with ultrasound or radiography, and reassurance that dose reduction does not eliminate this advantage is clinically important. Preserving the detection of other pathologies, such as appendicitis or vascular emergencies, supports the safety of implementing reduced-dose protocols as front-line imaging in acute flank pain presentations.

Using ROBIS, we judged one review to be at low risk of bias (Rodger et al. [27]), one at moderate risk (Drake et al. [25] due to heterogeneity and language restriction), and one at high risk (Rob et al. [26] due to absence of quality appraisal). These judgements temper confidence in the consistency of pooled estimates but do not alter the central message: across multiple systematic syntheses, reduced-dose CT KUB retains high diagnostic accuracy.

This overview is, to our knowledge, the first to collate and appraise all systematic reviews of reduced-dose CT KUB for renal colic. Evaluating methodological quality alongside diagnostic performance provides a higher-level perspective on the trustworthiness of existing evidence. We employed a comprehensive search strategy across four databases and followed best practice for selection and appraisal.

Nevertheless, limitations must be acknowledged. Only three systematic reviews met the inclusion criteria, and each drew on a relatively small pool of primary studies, limiting the breadth of evidence. Heterogeneity in dose definitions, scanner technology, and reference standards complicates direct comparison across reviews. Restriction to English-language reviews may have excluded relevant evidence from non-English publications. Finally, as this is an overview, we were dependent on the methods and reporting of the included reviews, some of which were incomplete.

The findings of this overview support the clinical adoption of LD and ULD CT KUB as first-line imaging for suspected renal colic in the majority of patients. Diagnostic performance is high, radiation burden is substantially reduced, and the ability to detect alternative diagnoses is largely preserved. Clinicians should, however, be aware of limitations in obese patients and for very small calculi, where diagnostic sensitivity may be reduced. In such cases, consideration may be given to standard-dose CT or tailored protocols depending on clinical suspicion and patient characteristics.

Future research should focus on standardising definitions of LD and ULD protocols, optimising techniques for obese patients, and evaluating clinical outcomes of reduced-dose imaging strategies, not just diagnostic accuracy. High-quality, multicentre prospective studies using contemporary scanner technology and iterative reconstruction are particularly needed. Moreover, further systematic reviews should apply best-practice methods for diagnostic test accuracy reviews, including bivariate meta-analysis, exploration of heterogeneity, and formal assessment of publication bias.

## Conclusions

This overview demonstrates that reduced-dose CT KUB provides high diagnostic accuracy for urinary tract stones, with pooled sensitivities and specificities exceeding 90% and results broadly consistent across systematic reviews. This is important given the fact that younger patients are often affected with acute renal colic, and minimising the radiation exposure is key, given the longer life expectancy in which radiation-induced malignancy may present, especially given the substantial risk of recurrence. The more widespread use of low-dose radiation protocols demonstrates progress in the domain of patient safety in radiology. Diagnostic performance is modestly reduced in obese patients and for very small stones but remains clinically acceptable in most contexts. The evidence base supports the use of LD and ULD CT KUB as reliable, radiation-sparing alternatives to standard-dose protocols in the investigation of suspected renal colic.

## Appendices

Search strings

**Table 3:** Ovid Medline search strings

Number	Search term
1	(non-contrast CT KUB or (non-contrast Computed Tomography of Kidneys, Ureters, and Bladder) or (non-contrast CT kidneys, ureters and bladder) or CT KUB Plain scan or (non-contrast Computed Tomography of Kidneys, Ureters, and Bladder Plain scan) or CT KUB without contrast agent or (Computed Tomography of Kidneys, Ureters, and Bladder without contrast agent) or NCCT KUB or non-contrast CT scan of the KUB region or (non-contrast CT scan of the Kidneys, Ureters and Bladder region) or (Non-contrast computeri*ed tomography scan of the kidneys, ureters and bladder) or Non-contrast computeri*ed tomography scan of the KUB or Unenhanced CT KUB or (Unenhanced Computed Tomography of Kidneys, Ureters, and Bladder) or Non-contrast CT of the urinary tract or Non-contrast Computed Tomography of the urinary tract or CT KUB without contrast or plain CT KUB or (plain Computed Tomography of Kidneys, Ureters, and Bladder) or native CT KUB or (native Computed Tomography of Kidneys, Ureters, and Bladder)).ti,ab,kw,kf
2	(Low dose or low dosage or small dose or small dosage or low quantity or low amount).ti,ab,kw,kf
3	(standard dose or Standard dosage or usual dose or usual dosage or typical dose or typical dosage or normal dose or normal dosage or recommended dose or recommended dosage or conventional dose or conventional dosage or regular dose or regular dosage or routine dose or routine dosage or accepted dose or accepted dosage or prescribed dose or prescribed dosage).ti,ab,kw,kf	
4	(Renal colic or Acute Renal Colic or Ureteral Colic or Kidney stone pain or Ureteric colic or Nephrolithiasis pain or Renal stone pain or Urolithiasis pain or Flank pain or Acute renal pain).ti,ab,kw,kf	
5	exp Renal Colic
6	4 or 5
7	1 or 2
8	1 or 7
9	6 and 7
10	6 and 8
11	9 and 10

**Table 4:** OVID Embase search strings

Number	Search term
1	(non-contrast CT KUB or (non-contrast Computed Tomography of Kidneys, Ureters, and Bladder) or (non-contrast CT kidneys, ureters and bladder) or CT KUB Plain scan or (non-contrast Computed Tomography of Kidneys, Ureters, and Bladder Plain scan) or CT KUB without contrast agent or (Computed Tomography of Kidneys, Ureters, and Bladder without contrast agent) or NCCT KUB or non-contrast CT scan of the KUB region or (non-contrast CT scan of the Kidneys, Ureters and Bladder region) or (Non-contrast computeri*ed tomography scan of the kidneys, ureters and bladder) or Non-contrast computeri*ed tomography scan of the KUB or Unenhanced CT KUB or (Unenhanced Computed Tomography of Kidneys, Ureters, and Bladder) or Non-contrast CT of the urinary tract or Non-contrast Computed Tomography of the urinary tract or CT KUB without contrast or plain CT KUB or (plain Computed Tomography of Kidneys, Ureters, and Bladder) or native CT KUB or (native Computed Tomography of Kidneys, Ureters, and Bladder)).ti,ab,kw,kf	
2	(Low dose or low dosage or small dose or small dosage or low quantity or low amount).ti,ab,kw,kf	
3	exp low drug dose/
4	2 or 3
5	(standard dose or Standard dosage or usual dose or usual dosage or typical dose or typical dosage or normal dose or normal dosage or recommended dose or recommended dosage or conventional dose or conventional dosage or regular dose or regular dosage or routine dose or routine dosage or accepted dose or accepted dosage or prescribed dose or prescribed dosage).ti,ab,kw,kf	
6	exp recommended drug dose/	
7	5 or 6
8	(Renal colic or Acute Renal Colic or Ureteral Colic or Kidney stone pain or Ureteric colic or Nephrolithiasis pain or Renal stone pain or Urolithiasis pain or Flank pain or Acute renal pain).ti,ab,kw,kf
9	exp kidney colic/
10	8 or 9
11	1 or 4
12	1 or 7
13	10 and 11
14	10 and 12
15	13 and 14

**Table 5:** PubMed search strings

Number	Search term
1	(non-contrast CT KUB or (non-contrast Computed Tomography of Kidneys, Ureters, and Bladder) or (non-contrast CT kidneys, ureters and bladder) or CT KUB Plain scan or (non-contrast Computed Tomography of Kidneys, Ureters, and Bladder Plain scan) or CT KUB without contrast agent or (Computed Tomography of Kidneys, Ureters, and Bladder without contrast agent) or NCCT KUB or non-contrast CT scan of the KUB region or (non-contrast CT scan of the Kidneys, Ureters and Bladder region) or (Non-contrast computerised tomography scan of the kidneys, ureters and bladder or Non-contrast computerized tomography scan of the kidneys, ureters and bladder ) or Non-contrast computeri*ed tomography scan of the KUB or Unenhanced CT KUB or (Unenhanced Computed Tomography of Kidneys, Ureters, and Bladder) or Non-contrast CT of the urinary tract or Non-contrast Computed Tomography of the urinary tract or CT KUB without contrast or plain CT KUB or (plain Computed Tomography of Kidneys, Ureters, and Bladder) or native CT KUB or (native Computed Tomography of Kidneys, Ureters, and Bladder)).
2	((Low dose or low dosage or small dose or small dosage or low quantity or low amount) OR ("low dose"[All Fields])) OR ("low drug dose"[All Fields])
3	(standard dose or Standard dosage or usual dose or usual dosage or typical dose or typical dosage or normal dose or normal dosage or recommended dose or recommended dosage or conventional dose or conventional dosage or regular dose or regular dosage or routine dose or routine dosage or accepted dose or accepted dosage or prescribed dose or prescribed dosage)
4	"standard dose"[All Fields]
7	#1 or #2
8	#1 or #4
9	((Renal colic or Acute Renal Colic or Ureteral Colic or Kidney stone pain or Ureteric colic or Nephrolithiasis pain or Renal stone pain or Urolithiasis pain or Flank pain or Acute renal pain) OR ("renal colic"[All Fields] OR "renal colic admissions"[All Fields])) OR ("renal colic"[All Fields])
10	#7 and #9
11	#8 and #9
12	#10 and #11

**Table 6:** Cochrane search strings

Number	Search term
1	non-contrast CT KUB or (non-contrast Computed Tomography of Kidneys, Ureters, and Bladder) or (non-contrast CT kidneys, ureters and bladder) or CT KUB Plain scan or (non-contrast Computed Tomography of Kidneys, Ureters, and Bladder Plain scan) or CT KUB without contrast agent or (Computed Tomography of Kidneys, Ureters, and Bladder without contrast agent) or NCCT KUB or non-contrast CT scan of the KUB region or (non-contrast CT scan of the Kidneys, Ureters and Bladder region) or (Non-contrast computeri*ed tomography scan of the kidneys, ureters and bladder) or Non-contrast computeri*ed tomography scan of the KUB or Unenhanced CT KUB or (Unenhanced Computed Tomography of Kidneys, Ureters, and Bladder) or Non-contrast CT of the urinary tract or Non-contrast Computed Tomography of the urinary tract or CT KUB without contrast or plain CT KUB or (plain Computed Tomography of Kidneys, Ureters, and Bladder) or native CT KUB or (native Computed Tomography of Kidneys, Ureters, and Bladder))
2	(Low dose or low dosage or small dose or small dosage or low quantity or low amount)
3	standard dose or Standard dosage or usual dose or usual dosage or typical dose or typical dosage or normal dose or normal dosage or recommended dose or recommended dosage or conventional dose or conventional dosage or regular dose or regular dosage or routine dose or routine dosage or accepted dose or accepted dosage or prescribed dose or prescribed dosage
4	(Renal colic or Acute Renal Colic or Ureteral Colic or Kidney stone pain or Ureteric colic or Nephrolithiasis pain or Renal stone pain or Urolithiasis pain or Flank pain or Acute renal pain)
5	MeSH descriptor: [Renal Colic] explode all trees
6	#4 or #5
7	#1 or #2
8	#1 or #3
9	#6 and #7
10	#6 and #8
11	#9 and #10
